# Locomotor Sub-functions for Control of Assistive Wearable Robots

**DOI:** 10.3389/fnbot.2017.00044

**Published:** 2017-09-04

**Authors:** Maziar A. Sharbafi, Andre Seyfarth, Guoping Zhao

**Affiliations:** ^1^Electrical and Control Engineering, School of Engineering, University of Tehran Tehran, Iran; ^2^Lauflabor Locomotion Laboratory, Institute of Sport Science, Centre for Cognitive Science, Technische Universität Darmstadt Darmstadt, Germany

**Keywords:** legged locomotion, locomotor sub-functions, stance leg control, swing leg adjustment, posture control, assistive devices, wearable robots

## Abstract

A primary goal of comparative biomechanics is to understand the fundamental physics of locomotion within an evolutionary context. Such an understanding of legged locomotion results in a transition from copying nature to borrowing strategies for interacting with the physical world regarding design and control of bio-inspired legged robots or robotic assistive devices. Inspired from nature, legged locomotion can be composed of three locomotor sub-functions, which are intrinsically interrelated: **Stance**: redirecting the center of mass by exerting forces on the ground. **Swing**: cycling the legs between ground contacts. **Balance**: maintaining body posture. With these three sub-functions, one can understand, design and control legged locomotory systems with formulating them in simpler separated tasks. Coordination between locomotor sub-functions in a harmonized manner appears then as an additional problem when considering legged locomotion. However, biological locomotion shows that appropriate design and control of each sub-function simplifies coordination. It means that only limited exchange of sensory information between the different locomotor sub-function controllers is required enabling the envisioned modular architecture of the locomotion control system. In this paper, we present different studies on implementing different locomotor sub-function controllers on models, robots, and an exoskeleton in addition to demonstrating their abilities in explaining humans' control strategies.

## 1. Introduction

**Figure 1:**
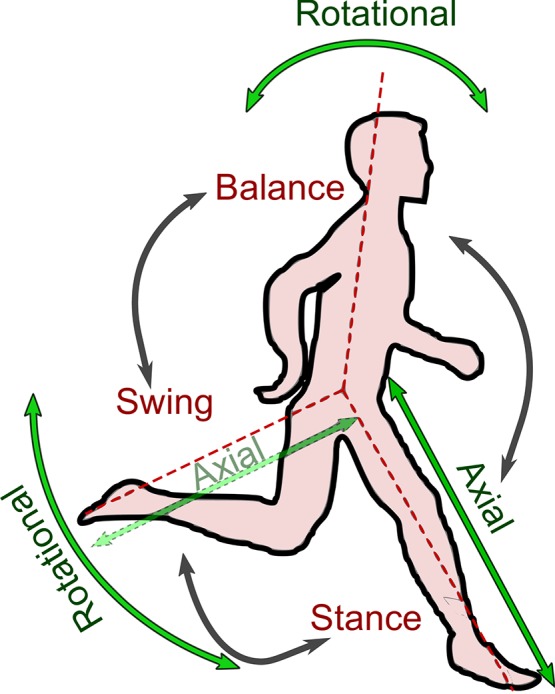
Main locomotion sub-functions; (i) axial stance leg function, (ii) rotational swing leg function, and (iii) balance for maintaining posture.

Unlike man-made vehicles, legged systems are the preferred biological technology for locomotion on ground. Research on legged locomotion, both in nature and robotics, helps us design and construct more agile and efficient moving systems. At the same time, it also supports understanding of human movement and control. In turn, this may help develop new approaches for locomotor rehabilitation and assistance. In this respect, findings in biology and robotics can greatly complement each other (Collins et al., 2015). Currently, the principles of animal and human locomotion and their applicability to artificial legged and assistive devices are not fully understood. Given the differences between biological and artificial body design and control, an important question is to what extent should we use biological design and control approaches for building artificial locomotor systems? Learning from nature does not require mimicking the biological locomotor system in detail. We can already greatly benefit of applying selected design and control principles, such as adding compliant structures to artificial systems or by arranging actuators analogous to bi-articular muscles in the human leg. In recent years, researchers from highly diverse disciplines such as biology, motion science, medicine and engineering have advanced research on legged locomotion by investigating underlying principles of body mechanics and related control design (Raibert, 1986; Duysens et al., 2002; Alexander, 2003; Holmes et al., 2006; Westervelt et al., 2007; Winter, 2009; Chevallereau et al., 2013). Considering nature as an ingenious teacher, bio-inspired approaches have become increasingly important in the study of legged locomotion (Duysens et al., 2002; Koditschek et al., 2004; Ijspeert, 2008). Legged locomotion can be composed of three locomotor sub-functions (Seyfarth et al., 2013): Stance (axial leg function), leg swinging and balancing, (Figure 1). Stance describes the elastic rebounding of the stance leg (ground contact) to counteract gravity (Blickhan, 1989). Leg swinging is mainly a rotational movement of the swing leg (Blum et al., 2010) combined with a minor axial leg movement for ground clearance. Since a major part of the body mass is located on the upper body, the human body is inherently unstable (Winter, 1995) and balancing (posture control, Massion, 1994) is considered to be a third locomotor sub-function, as a key feature of human gaits.

In this paper we explain how understanding bipedal locomotion using the concept of three locomotor sub-functions can be employed to design and control assistive wearable robots. In that respect, first, we survey our previous studies on combinations of the control concepts of three locomotor sub-functions to achieve stable gaits (Sharbafi et al., 2013b, 2014, 2016, 2017; Mohammadinejad et al., 2014; Oehlke et al., 2016; Sharbafi and Seyfarth, 2017; Zhao et al., 2017). Then, we show that considering such an architecture to control a wearable robot, an actuator can be designed with optimal performance for a range of motions required for different locomotor sub-functions. Therefore, a unifying actuation mechanism beside a bioinspired distributed control architecture can be employed to simplify interaction between different sub-functions and also between robot and human. It is noticeable that consistency between human and robot locomotion sub-function control not only facilitates interaction with humans, but also benefits from human movement control to orchestrate different sub-functions in the robot. For that, the controller for each sub-function needs to communicate with the related sub-function on human body through sensory feedback.

To implement the proposed distributed control architecture we employ the “Template & Anchor” concept (Full and Koditschek, 1999). Despite their high level of abstraction, template models are very useful tools to understand how these sub-functions are controlled and coordinated, both in nature (Blickhan, 1989) and legged robots (Raibert, 1986). In our studies we have applied mass-spring, physical and virtual pendulums as our template models for stance, swing and balance, respectively. Pendulum and mass-spring as two oscillators are very useful tools for explanation of legged locomotion as a rhythmic movement. Table 1 presents an overview of locomotor sub-function concept including basic characteristics and samples of representative template models. In the following, first we describe this concept including relevant template models in Section 2. Then, Section 3 explains how these sub-functions help better understand human gaits. In Section 4, different instances of implementation on models, robots and exoskeletons are presented. Finally, Section 5 discusses how one can benefit from the proposed concept in design and control of wearable robots to facilitate interaction with humans and to provide a more harmonized control of different sub-functions through actuators.

**Table 1:** Overview of locomotion sub-functions with basic characteristics and their representation in template models.

**Locomotion sub-function**	**Objective**	**Leg force direction**	**Biomechanical template models**
Stance	Interacting with ground	In leg axis	Leg spring
Swing	Adjust leg orientation during swing phase	Perpendicular to and in leg axis	Pendulum-like leg with adaptable length + Hip spring
Balance	Maintaining an upright body orientation	Perpendicular to leg axis	Hip spring, virtual pendulum

## 2. Locomotor sub-function concept and template models

Legged locomotion is a complex task with integrated functional levels influencing all three locomotor sub-functions. Our separate treatment of these sub-functions allows integration of key functional features at each level of legged locomotion (mechanics, actuation, sensing and control).

For stable legged locomotion, a control architecture is required to employ the locomotion concepts. Template models (Full and Koditschek, 1999) which present reduced order systems of the locomotors, are our tools to understand how the sub-functions are controlled and coordinated. We need to know the corresponding control concepts and to learn from biology to simplify control. In addition, for interaction with humans, lower level force/torque control is beneficial in comparison to position control which might be harmful for humans (Haddadin et al., 2008). We show that such template-based control approaches are founded on impedance (e.g., stiffness) control which consider this latter concern.

In order to benefit from bioinspired locomotion concepts that can be used for implementation on robots or assistive devices, key characteristics of legged mechanisms need to be identified. Based on realizing legged locomotion with the aforementioned trilogy, we have investigated different bioinspired control approaches on human experimental data, conceptual models and finally robots and exoskeletons, presented in the next two sections. Here, we describe locomotor sub-functions and their related template models.

The proposed models which are based on the concept of locomotor sub-functions are inspired from human locomotion. This concept is used for gait modeling that can be further extended for design and control of robots. Here, we propose to implement this technique to control exoskeletons (as wearable robots) which have interactions with humans. The key idea is using the bioinspired control techniques based on locomotor sub-function theory to make the control of the wearable robots (lower-body exoskeletons) compatible with human movement.

### 2.1. Relevant template models

#### 2.1.1. SLIP for stance

Stance function describes the repulsive function of the stance leg (in contact with the ground) to counteract gravity (Seyfarth et al., 2013). A spring-loaded inverted pendulum (SLIP) model (Blickhan, 1989) is a simple template model describing human-like axial leg function in walking and running (Geyer et al., 2006). In this model the force-length relationship of the leg in axial direction is approximated by a spring which is linear in running and hopping and nonlinear in walking. SLIP is popular for its ability in describing human gaits and modeling of legged robots. However, it can be also employed as a template for control e.g., Poulakakis and Grizzle (2009) and Wensing and Orin (2013). In such studies the linear force-length relationship of the virtual leg (a line between CoM and CoP) is utilized as a target for control. In this approach, stance leg control goal is developing joint torques to yield spring-like behavior of the virtual leg. In Section 4, we explain how this method is used in different applications: (1) Mimicking human-like leg elastic behavior with a robot (Sharbafi et al., 2014; Oehlke et al., 2016) e.g., implemented by VMC (Virual model control) (2) energy management through ankle torque and biarticular muscles (Sharbafi et al., 2014, 2016). There are also Extended SLIP models, like ESLIP (Ludwig et al., 2012) or the variable leg spring (VLS) model (Riese et al., 2013), describing leg spring adjustments (stiffness, rest length) during the stance phase. These models can be also implemented to achieve higher control performance. Since this approach is consistent with human stance leg control, it is expected to provide appropriate interaction between human and robot while applying this technique to control a wearable robot.

#### 2.1.2. Pendulum for swing

Leg swinging is mainly a rotational movement combined with a complementing axial leg movement to avoid foot scuffing on the ground. Regarding swing leg control, we follow two approaches: The first approach is an improvement of the Raibert leg adjustment approach (Raibert, 1986) using the CoM velocity to find the desired leg angle. This VBLA (velocity based leg adjustment) method (Sharbafi and Seyfarth, 2016) provides a stabilizing control strategy for different gaits (Sharbafi et al., 2013b, 2014; Sharbafi and Seyfarth, 2015) and also nicely describes human perturbation recovery for hopping in place (Sharbafi and Seyfarth, 2013). Although this can be employed to control the swing leg as one of the locomotor sub-functions, it is not at the focus of this paper because of lack of template model describing the control concept.

**Figure 2:**
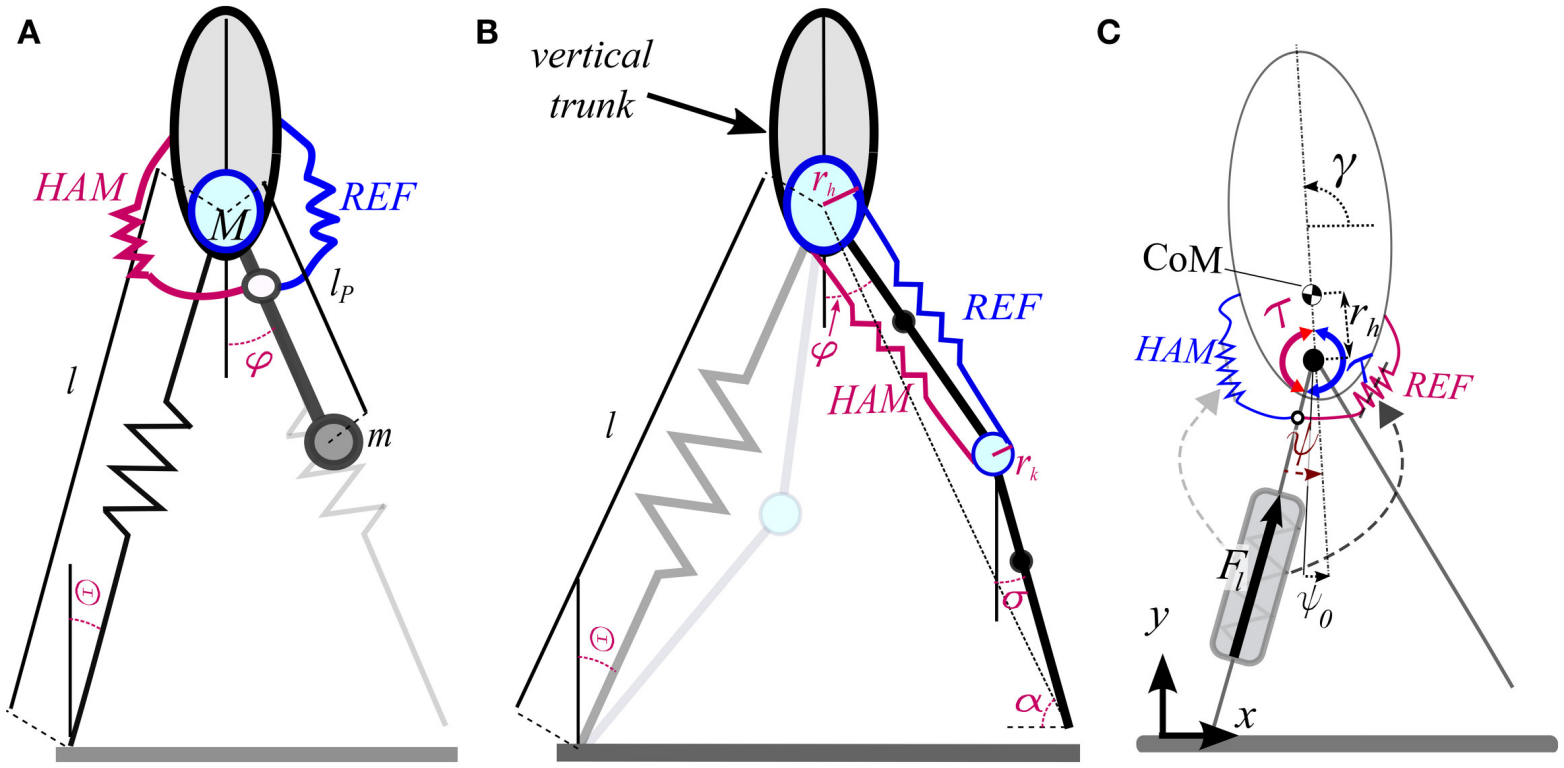
**(A)** SPS (Springy Pendulum SLIP) model of swing leg adjustment. **(B)** BDPS (Biarticular muscle equipped Double Pendulum SLIP) model for leg swinging. A virtual upright trunk is considered, from which the hip angle (φ) is computed in SPS and BDPS models. The spring of the stance leg can be replaced by any models mimicking SLIP like behavior (e.g., with segmented leg as shown by light colors). **(C)** FMCH for posture control in bipedal walking. In current state, the red spring is producing (negative) rotational torque as a hip flexor muscles (Rectus femoris or Iliopsoas) while the blue hip extensor spring (Biceps femoris or Gluteus Maximus) is slack.

Instead of Velocity based leg adjustment, a second approach which can be considered as a template based control method for leg swinging is to assume a passive pendulum-like movement of the swing leg (Knuesel et al., 2005; Mohammadinejad et al., 2014). Mochon and McMahon presented a model comprising a stiff stance leg and a segmented swing leg (Mochon and McMahon, 1980) which provides a better match of human walking dynamics, compared with the inverted pendulum model. Another improvement in modeling human gait dynamics (GRF and COM movement) was obtained by replacing stiff leg with massless spring in SLIP model (Geyer et al., 2006). However, swing leg movement is still a missing part in SLIP based models. In Mohammadinejad et al. (2014), we presented a new model combining SLIP for stance leg with pendulum movement for the swing leg in running. In this model the pendulum length is adapted at each step to attenuate any perturbation or error from desired movement. Adding hip rotational springs to this pendulum instead of pendulum length adaptation (see Figure 2A) result is SPS (Springy pendulum SLIP) model. In Section 3, we show that this model can precisely predict human swing leg adjustment.

In this model, we consider two decoupled dynamics for the stance and swing leg with Equation (1). This model is precise if the CoM moves horizontally (keeping the height) with constant speed. However, simulations show that this decoupled model can well approximate human swing leg movement as shown in Section 3.

(1) $$\left[\begin{array}{c} \ddot{l}\\ \ddot{\theta }\\ \ddot{\varphi } \end{array}\right]=\left[\begin{array}{c} \frac{k}{M}(l_{0}-l)-g\text{ cos}(\theta )+l{\dot{\theta }}^{2}\\ \frac{g}{l}\text{ sin}(\theta )-2\frac{\dot{l}\dot{\theta }}{l}\\ \frac{-g\text{ sin}(\varphi )}{l_{p}}+k_{REF}max(\varphi_{0}^{REF}-\varphi ,0)\\ -k_{HAM}max(\varphi -\varphi_{0}^{HAM},0) \end{array}\right]$$

in which *k*, *l*_0_ the stiffness and the rest length of the stance leg. In this equation we considered rotational springs to model the biarticular thigh muscles. For this, we use *k*_*REF*_ and *k*_*HAM*_ are the normalized stiffness of the rectus femoris and hamstrings muscles, respectively and $\varphi_{0}^{REF}$ and $\varphi_{0}^{HAM}$ are the rest angles for the related muscles. The rest of parameters can be found in Figure 2A. The *max* function guarantees that the muscles are unidirectional. The normalized stiffness is calculated by the following equations

(2) $$\{\begin{array}{l} k_{REF}=\frac{k_{REF}^{rot}}{ml_{p}^{2}}\\ k_{HAM}=\frac{k_{HAM}^{rot}}{ml_{p}^{2}} \end{array}$$

in which, *m*, $k_{REF}^{rot}$ and $k_{HAM}^{rot}$ are the pendulum (swing leg) mass, stiffness of rotational springs for REF and HAM muscles, respectively. Here, the relation between leg mass, the muscle stiffness and the pendulum length is ignored by normalizing the stiffness.

Touchdown happens when the swing leg hits the ground. The subsequent double support is described with the BSLIP model (Geyer et al., 2006). Here, we neglect the swing leg mass and therefore the impact effect at touchdown. Considering the same stiffness and rest angle for both hip muscles (*k*_*h*_ = *k*_*REF*_ = *k*_*HAM*_ and $\varphi_{0}^{h}=\varphi_{0}^{REF}=\varphi_{0}^{HAM}$) results in the following swing dynamics.

(3) $$\begin{array}{r} \ddot{\varphi }=\frac{-g\text{ sin}\left(\varphi \right)}{l_{p}}+k_{h}\left(\varphi_{0}^{h}-\varphi \right) \end{array}$$

In Section 3, we use Equation (1) to predict human swing leg angle and angular velocity during walking at different speeds.

In Sharbafi et al. (2017) this pendulum-based model is extended to a two segmented swing leg equipped with biarticular springs. This models is called BDPS standing for Biarticular muscle equipped Double Pendulum SLIP (Figure 2B). In the SPS model (Figure 2A) described by Equation (1) the first two rows explain the stance leg dynamics of the SLIP model and the last row describes the swing leg dynamics. In that respect the stance and swing leg dynamics are decoupled. In the BDPS model we consider coupling between stance and swing leg dynamics which may help better predict human motor control and produce more synchronized joint control in robots. There are many other extended models which can be used as templates for control of different locomotor sub-functions. For example OĆonnor introduced a new SLIP-based model with additional mass in both legs, curved feet and hip rotational spring (OĆonnor, 2009). However, here we focus on the simplest models that can represent the gait features required for control of the sub-functions.

Judging from human leg muscle activities in the swing leg movement, biarticular hip muscles; rectus femoris (REF) and hamstrings (HAM) seem to be the main contributors for swing leg control in walking (Nilsson et al., 1985). By modeling these two muscles with biarticular springs, better mechanical understanding of their activities in producing stable gait is obtained. In addition, such a passive mechanism may also replicate strong correlation observed between RF and HA in human swing leg movement (Prilutsky et al., 1998), as a consequence of body mechanics. The role of elastic biarticular thigh muscles (represented as springs) on swing leg dynamics can be further investigated, and the appropriate spring parameters and morphology can mimic human swing leg motion in walking. The muscle lever arm ratio, muscle stiffness and muscle rest lengths influence the CoM motion and the swing leg behavior. With passive elastic biarticular muscles, walking motion characteristics, like swing leg retraction and symmetric stance leg behavior around mid-stance are predicted (Sharbafi et al., 2017).

In this model, the double pendulum with biarticular springs for the swing leg can be combined with any model of stance leg (e.g., SLIP model). Here we describe BDP (Biarticular muscle equipped Double Pendulum) formulation for swing leg modeling while representing BDPS as an example to have SLIP for the stance leg to complete the walking model. Depending on the stance leg model, switching mechanisms between two legs at touchdown and takeoff needs to be defined. Let *q*_*s*_ be the configuration variables of the stance leg (e.g., $q_{s}={\left[\theta \;l\right]}^{T}$ for BDPS) and define $q={\left[q_{s}^{T}\;\varphi \;\sigma \right]}^{T}$ as depicted in Figure 2B (super index *T* shows transpose operator.). Then, the following dynamic equation of the model are obtained.

(4) $$\begin{array}{r} D\left(q\right)\ddot{q}+C\left(q,\dot{q}\right)\dot{q}+G\left(q\right)=F \end{array}$$

in which *G*, *D*, and *C* are the gravity vector, the inertia and the Coriolis matrices, respectively. The last two rows of the force vector (*F*) are calculated from summation of the hip and knee torques generated by REF and HAM springs (see below). The hip torque is exerted between the thigh and a virtual upright trunk which approximates the normal upper body posture in human locomotion as described in Maus et al. (2010). For this, posture control as the third locomotor sub-function will be addressed in the next section. Suppose the lever arms at hip and knee are described by *r*_*h*_ and *r*_*k*_, respectively. Then, the elongation of REF (from rest length) will be

(5) $$\begin{array}{r} \Delta l^{REF}=r_{k}\theta_{k}-r_{h}\varphi -l_{0}^{REF} \end{array}$$

in which θ_*k*_ = φ−σ and $l_{0}^{REF}$ are the knee angle and the REF spring rest length, respectively. The net torques generated by the REF acting at the thigh ($\tau_{\varphi }^{REF}$) and the shank ($\tau_{\sigma }^{REF}$) are computed as:

(6) $$\{\begin{array}{l} \tau_{\varphi }^{REF}=k_{REF}(r_{h}-r_{k})max(\Delta l^{REF},0)\\ \tau_{\sigma }^{REF}=k_{REF}r_{k}max(\Delta l^{REF},0) \end{array}$$

where *k*_*REF*_ is the stiffness of the REF spring. By antagonistic arrangement the hip and knee torques provided by the HAM can be calculated in a similar manner. In the BDPS model that uses spring for stance leg generating leg force *F*_*s*_, the force vector *F* will be calculated as follows.

(7) $$F=\left[\begin{array}{c} 0\\ F_{s}\\ \tau_{\varphi }^{REF}+\tau_{\varphi }^{HAM}\\ \tau_{\sigma }^{REF}+\tau_{\sigma }^{HAM} \end{array}\right]$$

Such a simple bioinspired control approach can be easily implemented in robots (Sharbafi et al., 2016). During the swing phase, biarticular muscles can support swing leg rotational movement control while monoarticular muscles (e.g., knee or ankle joints) can provide (axial) leg shortening and lengthening (e.g., leg shortening is required for ground clearance). With such a muscle-specific task allocation, the target of control could be simply setting spring rest lengths to a specific value for each gait condition. Considering Variable Impedance actuators (VIA) as tunable compliant elements, such passive leg swinging methods can be mimicked by bi-articular VIAs. In Section 4 the results of applying this model for control of leg swinging sub-function in BioBiped robot are presented.

#### 2.1.3. Virtual pendulum for balance

Humans and other bipeds unlike quadrupeds need to take care of their upper bodies to avoid falling. Since there is no upper body in SLIP model, it can not describe posture control. In other words, one of the shortcomings of the SLIP model is its inability in predicting ground reaction forces (GRF) direction while it is always intersecting CoM. In contrast, in the stance phase of (upright) human walking, the GRFs are intersecting in a virtual pivot point (VPP, Maus et al., 2010) or divergent point (DP, Gruben and Boehm, 2012) above the center of mass (CoM). Therefore, the SLIP model needs to be extended by a segment (e.g., rigid trunk) representing the upper body (e.g., TSLIP model for Trunk SLIP). Based on VPP concept, postural balance control can be understood as converting the upright inverted (body) pendulum into the regular pendulum model. In this model a virtual pendulum (VP) can be defined with a point mass at CoM hanging from the VPP. From a control point of view, this concept can be employed to derive balancing strategies.

From another point of view, having appropriate controllers for two other locomotor sub-functions, a stable gait is achievable by producing a hip torque to redirect the ground reaction forces to a predefined VPP (Maus et al., 2010; Sharbafi et al., 2012). In an extension of the model, the VPP is adjusted at each step (called virtual pendulum posture control, VPPC), which results in robust hopping (Sharbafi et al., 2013b). Surprisingly, the desired control performance can be partially achieved by an appropriate hip compliance design, as can be seen in Rummel and Seyfarth (2010) and Sharbafi et al. (2013a). However, to achieve more human-like hip torque control and better matching to VPP concept, a reflex signal representing leg force is needed to adjust the hip compliance (Sharbafi and Seyfarth, 2014). As a result, a new model called FMCH (force modulated compliant hip) can physically implement the VPP concept with a neuro-muscular structure (Figure 2C). In this model, the hip torque between the virtual leg and the upper-body is generated by an adaptable spring. The virtual leg is defined from CoP to the hip. The hip torque τ is given by

(8) $$\begin{array}{r} \tau =k_{REF}max\left(\psi_{0}^{REF}-\psi ,0\right)-k_{HAM}max\left(\psi -\psi_{0}^{HAM},0\right). \end{array}$$

in which, ψ, ψ_0_ and *k* are the trunk to leg angle, rest angle and stiffness of the hip spring, respectively. The super-/sub-index *REF* and *HAM* indicate the corresponding muscle. First, we assume the same stiffness (*k*_*h*_ = *k*_*REF*_ = *k*_*HAM*_) and rest angle ($\psi_{0}=\psi_{0}^{HAM}=\psi_{0}^{HAM}$) for both hip springs. Then, the stiffness of this rotational spring (*k*_*h*_) is adjusted using the leg force *F*_*s*_ feedback as follows:

(9) $$\begin{array}{r} \tau =k_{h}\left(\psi_{0}-\psi \right)=cF_{s}\left(\psi_{0}-\psi \right) \end{array}$$

In this formulation, the hip stiffness *k*_*h*_ is given by leg force *F*_*s*_ multiplied by a constant value *c*. In addition to benefiting from motion dynamics to synchronize the locomotor sub-functions (like in the BDPS model), here a feedback from one sub-function (leg force from stance) to another (hip compliance controlling balance) improves coordination between locomotor sub-functions for generating a stable gait. This yields a clear VPP above CoM in the upper body coordinate system (Sharbafi and Seyfarth, 2014).

This approach results in stable walking (Sharbafi and Seyfarth, 2015) and running (Sharbafi and Seyfarth, 2014) as predicted by the model. FMCH model represented by Equation (6) not only can describe human posture control (Sharbafi and Seyfarth, 2017), but also can be easily implemented on robots (Sharbafi et al., 2016) and exoskeletons (Zhao et al., 2017). The outcomes of such implementations are described in Section 4. Interestingly, it was found that the combination of locomotor sub-functions based on implicit coordination (with a limited exchange of sensory information) can produce stable gaits e.g., forward hopping (Sharbafi et al., 2014, 2016). This supports the idea of implementing separate sub-function controllers in wearable robots.

### 2.2. Human experiment

Two experiments are employed to verify the locomotor sub-function theory in this paper. The first data set was collected in walking experiments on a treadmill (type ADAL-WR, Hef Tecmachine, Andrezieux Boutheon, France) at different speeds. Motion capture data and ground reaction force data are measured by Qualisys setup (Gothenburg, Sweden) from 11 markers and from force sensors within the treadmill, respectively. Twenty one subjects (11 female, 10 male, age: 22–28 yrs, height: 1.64–1.82 m, weight: 59.2–82.6 kg) were asked to walk at different percentages of their preferred transition speeds (PTS)^1^. The treadmill speed was employed as the walking speed. Kinematic and kinetic data processing was described in Lipfert (2010).

In the second experiment, a newly developed FMCH control approach is implemented on an assistive wearable robot (LOPES II) for slow walking (around 25% *PTS*). The experiment protocol includes three phases: (i) Initiation: In order to be familiarize with the exoskeleton, each subject had a test walking trial (about 3–5 min) wearing the exoskeleton. (ii) Warm-up: It is 3 min walking with the robot in transparent mode for warming up (without data collection), (iii) Data measurement: 7 min assisted walking, 7 min transparent walking, and 3 min quiet standing for measuring the bias values.

This study was carried out in accordance with the recommendations of “CCMO (Central Committee on Research Involving Human Subjects) consent procedure” with written informed consent from all subjects. All subjects gave written informed consent in accordance with the Declaration of Helsinki. The protocol was approved by the “METC Twente (local ethical committee).”

More details about this experiment are described in Section 4 and Zhao et al. (2017).

## 3. Human gait

In Sharbafi and Seyfarth (2017), different sub-function models are utilized to predict human control strategies for walking at different speeds. The SLIP model with adaptable leg stiffness and rest length which are changing at middle of single support was used to predict the stance leg force. It was shown that the leg spring behavior is clearly changing at the middle of swing phase. The higher performance of SLIP model with a variable spring (compared to the fixed spring) in energy management and perturbation recovery were also depicted in Ludwig et al. (2012) and Sharbafi et al. (2013b), respectively. Such a method can be implemented to control robots and assistive devices. In the next section we explain how to use this approach for control of (wearable) robots.

In the aforementioned study of different sub-functions' roles in speed adjustment, the angle of attack and the angular speed at touchdown moment were used to characterize the swing leg control (Sharbafi and Seyfarth, 2017). In addition, the VBLA controller was also compared to other methods regarding their abilities in explaining human leg adjustment. However, these are not the template model to be used for the second locomotor sub-function. Furthermore, none of the above methods can control the swing leg continuously during swing phase and they mostly aim at finding an appropriate angle of attack. Here, we utilize the two template models of swing leg control presented in previous section (see Figure 2A for SPS and Figure 2B for BDPS).

**Figure 3:**
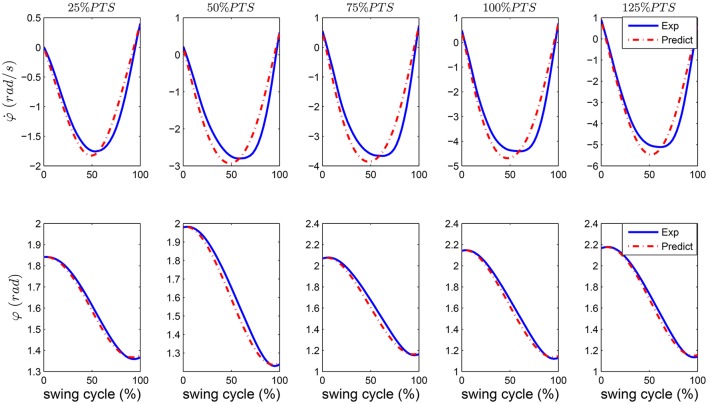
The swing leg angles and angular velocities in human walking experiment (Exp) are shown by blue solid line and predictions of these values with the SPS model (Predict) are shown by red dash-dotted lines.

Using the SPS model we predict the patterns of human leg angle and angular velocity changes in walking at different speeds as shown in Figure 3. The blue curves show experimental data and the red dash-dotted curves demonstrate the prediction of the SPS model. In these graphs we use the initial leg angle (φ(0)) and angular speed (($\dot{\varphi }\left(0\right)$) at takeoff and calculate the acceleration from experimental data and the swing trajectories ($\left[\varphi \left(t\right)\;\dot{\varphi }\left(t\right)\right]$) using Equation (1). Implementation of this approach for control needs just the current leg angle for computing the required torque to generate the desired acceleration. The quality of prediction by the SPS model is shown in Table 2 using *R*^2^ index for correlation. It can be seen that the precision of predicting the leg angle during swing phase is 0.98 or 0.99 at different speeds.

**Table 2:** Precision of predicting human swing leg movement during single support of walking, with SPS model.

**Walking speed**	**25%PTS**	**50%PTS**	**75%PTS**	**100%PTS**	**125%PTS**
Leg angle	0.99	0.98	0.98	0.99	0.99
Angular speed	0.94	0.87	0.89	0.91	0.93

*Correlation between prediction and real values are shown by R^2^ values*.

Our another template for swing leg is the so called BDPS model in which the swing leg is modeled by a double pendulum equipped with biarticular springs. In Sharbafi et al. (2017), it was shown that using this model stable walking can be achieved without energy consumption for leg swinging, while energy injection is performed by tuning the springs rest lengths just before takeoff. Using SLIP for modeling the stance leg, we have shown that appropriate tuning of the biarticular springs' rest lengths are sufficient for swing leg adjustment. Similarity between muscle force patterns of the BDPS model and human subjects were demonstrated in Sharbafi et al. (2017). Therefore, if the posture control sub-function can perfectly keep the upper body upright, the leg swinging strategy beside spring-like behavior of the stance leg (with appropriate stiffness and rest length) results in a stable gait.

For the third locomotor subfunction, the hip torques in the single support of human walking at different speeds were predicted by the FMCH model with sufficiently high precision. Hence, this model can be used as our template model for posture control. Considering the FMCH for posture control beside BDPS or SPS for swing leg and adjustable spring for stance leg can generate a stable model of locomotion which can explain human walking features precisely. In Section 4, we demonstrate how these models can be used for control of different sub-functions in isolation and in collaboration.

## 4. Implementation

Our template for stance leg control is the SLIP model, either with fixed stiffness for bouncy gaits, such as hopping and running or variable stiffness for walking. In a pilot research about control of knee actuator for lower limb exoskeleton, a segmented leg is developed that moves in vertical direction. This robot, called MARCO-Hopper-II, was the next generation of MARCO-Hopper developed 10 years ago (Seyfarth et al., 2007). In studies on MARCO-Hopper, the motor torque was simulating either a linear leg spring (based on SLIP model) or a muscle-reflex system. For stable hopping, significant energy supply was required after mid-stance, achieved by enhancing leg stiffness (Kalveram et al., 2012) or by continuously applying positive force feedback (Seyfarth et al., 2007). In Oehlke et al. (2016), we have implemented the SLIP-based stance leg control on the MARCO-Hopper-II robot to mimic human hopping in place. The virtual model control (VMC) (Pratt et al., 2001) and energy-management (Kalveram et al., 2012) were two approaches to implement this control strategy on the robot. Stable hopping with similar features to human hopping was achieved using SLIP as the template for control. Similar to findings in human gaits, changing the stiffness of the virtual spring (between the hip and the foot) is required to control the robot for energy management. The hardware is to be extended with addition of spring in series with the electric motor as the next step of developing human-like motor control for assistive devices. Employing SEAs (series elastic actuators) for control of wearable robots is beneficial as can be seen in Walsh et al. (2006) and Eslamy et al. (2012).

**Figure 4:**
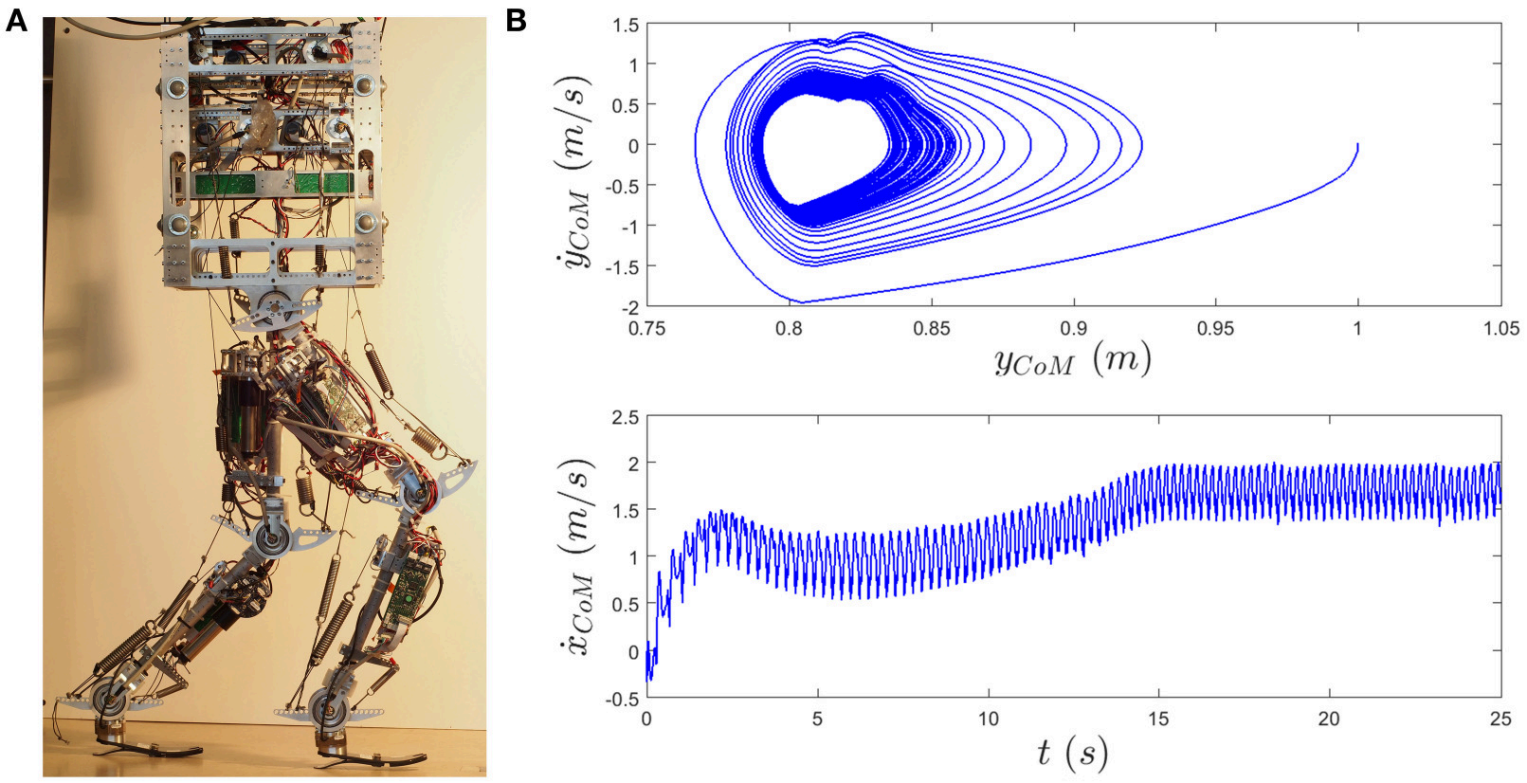
**(A)** BioBiped 3 robot. **(B)** The results of forward hopping with BioBiped detailed model based on locomotor sub-function control concept. Top figure shows the limit cycle (*y*_*CoM*_ is the CoM height) and bottom figure illustrates the forward speed of the CoM (ẋ_*CoM*_). The template model for control is the BDPS model. The speed is adjusted using the stiffness of stance leg spring through ankle joint control and the rest lengths of the biarticular thigh springs.

In Sharbafi et al. (2014), a similar approach for stance leg control was implemented on the detailed simulation model of BioBiped robot (Figure 4A) for forward hopping. In Figure 4A, the third version of this robot series, called Biobiped 3, is shown (see Sharbafi et al., 2016 and www.biobiped.de for more information). In addition to apply VMC for SLIP-based control of the stance leg, we employed the VBLA (Sharbafi and Seyfarth, 2016) for swing leg adjustment. Stable forward hopping was achieved with this combination of two sub-functions while the upper body was balanced using mechanical constraints (Sharbafi et al., 2014).

Combination of different locomotor sub-functions (stance and swing) is tested on the detailed model of the robot using BDPS template model. Here, we use SLIP for control of the stance leg and the thigh biarticular actuators (muscles) for swing leg adjustment (see Section 2.1.2 for details). Stable forward hopping with adjustable horizontal speed is achieved using this technique. Figure 4B shows the results of speed adjustment and the stable limit cycle in vertical direction. The simulation starts by dropping the robot from 1 m height. As shown in the bottom figure, the controller increases the robot speed to reach 1 m/s horizontal speed and hops with this speed for 3 s (from 5 to 8 s). Then, by readjustment of the rest length of swing leg thigh springs and the stiffness of the virtual stance leg using the ankle joint, the speed increases to 1.75 m/s. The contributions of the stance and swing leg controllers are different for the first and the second commanded speeds. This demonstrates the ability of the proposed controller to consider different features in tracking a desired input. As a result, the small overshoot in reaching 1 m/s is not observed in the second acceleration phase to set the speed to 1.75 m/s, whereas the settling time is smaller in response to the first commanded speed. Here, the role of the stance control (injecting a fixed amount of energy through SLIP-based controller using VMC) is more significant in the first phase (1 m/s) compared to second phase (1.75 m/s), while this is other way around for the swing leg control (adjusting the thigh biarticular muscles' rest lengths). This is implemented by larger relative changes (from first to the second phase) in swing leg control than stance control. Therefore, when the contribution of the stance leg control is higher, the response is faster including overshoot, whereas a higher contribution of swing leg control results in slower convergence without overshoot.

The top figure illustrates the phase plot in the space of CoM height *y*_*CoM*_ and vertical speed ẋ_*CoM*_. It shows that after reaching a certain forward speed the phase trajectories converge to a stable limit cycles corresponding to hopping horizontal speed. In addition, with increasing the motion speed, the shape of the limit cycle is fixed, whereas the size shrinks.

These results show that simple controller based on the locomotion sub-function concept can properly stabilize the robot motion. Note that the upper-body is playing the role of the coordinator between the two sub-functions. Here the posture control is exerted by enforcing physical constraints and there is no need for further exchange of sensory information. The same concept can be employed in control of exoskeletons while human posture control can be complemented by assistive swing and stance leg control from the robot.

**Figure 5:**
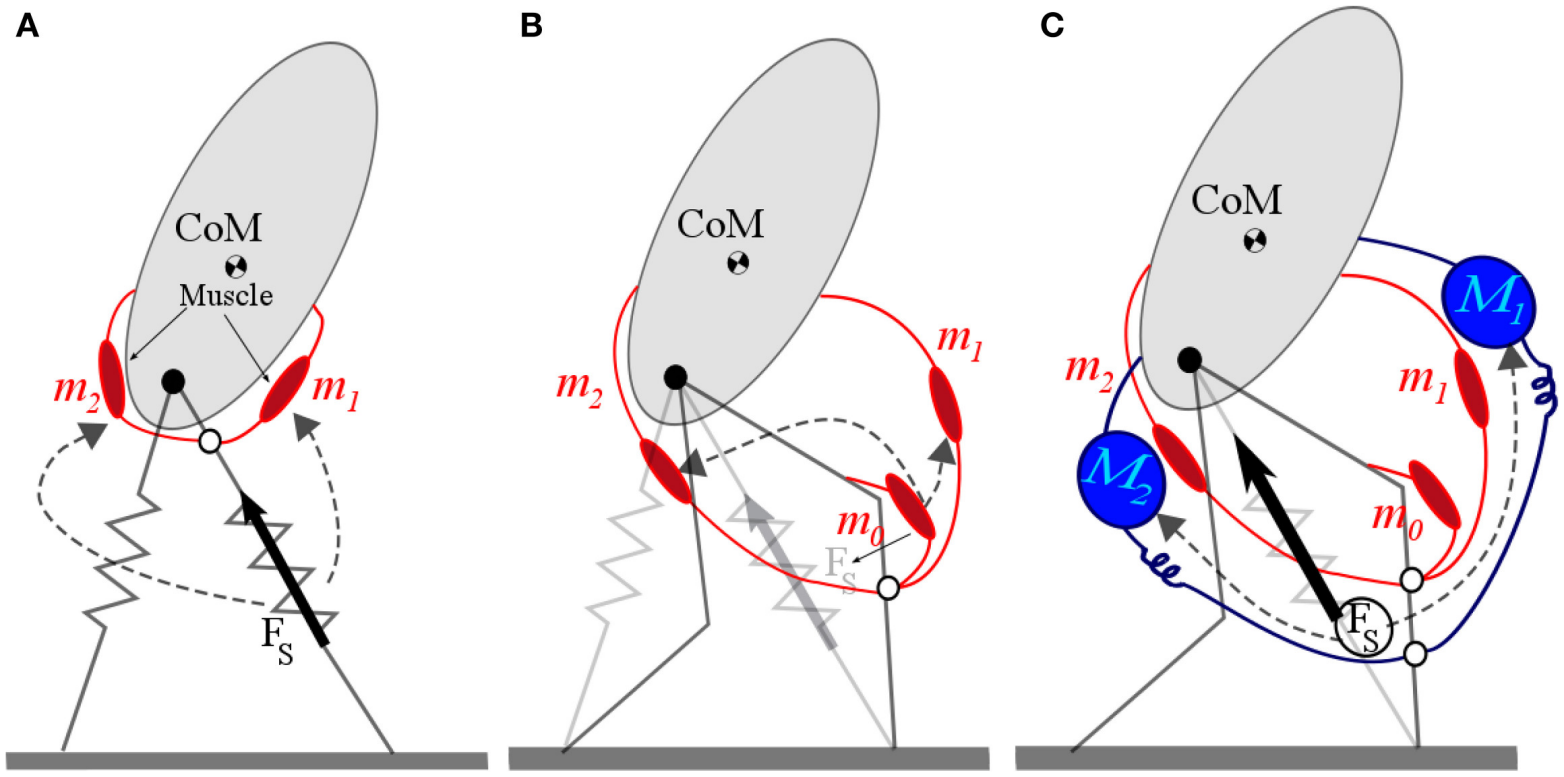
Steps of extending FMCH model to neuromuscular level and exoskeleton control. **(A)** Replacement of hip spring with muscle model, **(B)** segmentation of the leg and replacement of the leg spring by knee extensor muscle, **(C)** implementation of assistive control in addition to muscle activation.

Based on the bioinspired VPP (virtual pivot point) concept, introduced in Maus et al. (2010), we have developed the FMCH model, in which adaptable hip springs are considered for balancing while the spring stiffness is modulated by leg force. We have shown that this control approach results in a VPP using conceptual models (Sharbafi and Seyfarth, 2015). Successful addition of posture control to aforementioned stance and swing leg controllers were shown with different combinations of sub-function controllers for different gaits (Sharbafi et al., 2013b,a; Sharbafi and Seyfarth, 2014). Recently, we have examined the idea of distributed control of sub-functions in two new directions: extending the template models to neuromuscular level and implementing the controllers on robots. In Figure 5 we showed th required steps. In the first step, the rotational hip springs are replaced by muscle models as shown in Figure 5A. With this model we could achieve stable walking with GRFs and hip torques similar to FMCH model (respectively to human gaits). Afterward, we substituted the leg spring with a two segmented leg including the knee extensor muscle (Figure 5B) and could achieve stable walking. Although the patterns are not completely similar to previous models, the VPP exists which shows the consistency of the model with previous models and also to human posture control. The last step is using this model for human walking and emulate the exoskeleton via addition of actuators (e.g., SEAs) to assist human walking as shown in Figure 5C. With this model using the FMCH-based controller, a method for control of the exoskeleton (e.g., wearable robot) is developed which is in line with the neuromuscular control of humans. The details of these simulation studies are out of scope of this paper and will be presented elsewhere. Here, we explain the results of our recent implementation of this method on a wearable robot.

**Figure 6:**
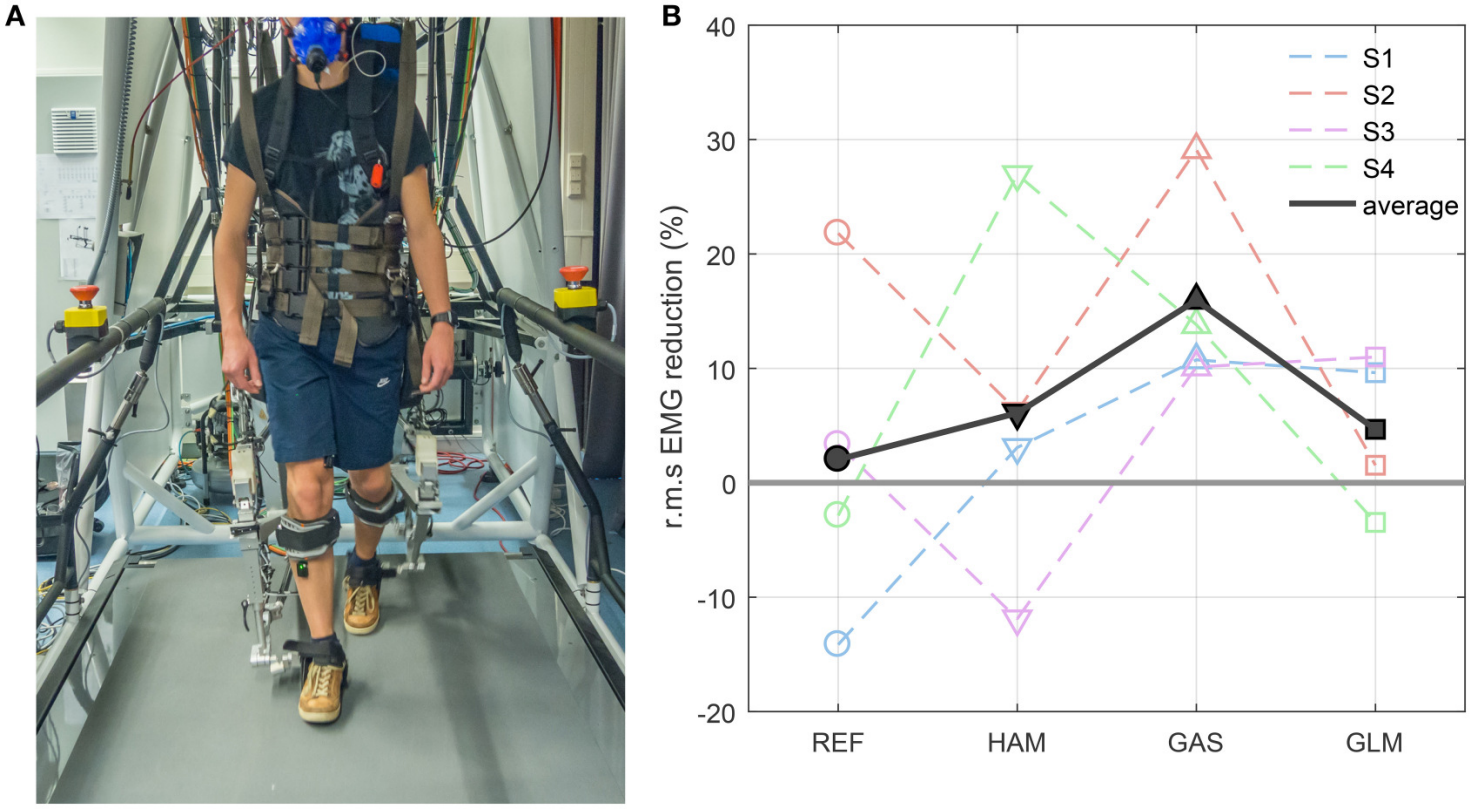
**(A)** Experiment with LOPES II, **(B)** reduction in root mean square (r.m.s) of muscle activations for different subjects shown by different colors and dashed lines. The black solid line depicts the average of the EMG reduction for the four subjects. S1 to S4 indicate the subjects number.

In this experiment we have implemented an FMCH-based controller on a lower limb powered exoskeleton (LOPES II) and demonstrated that it can effectively assist humans during walking. The experiment is performed with four young healthy subjects (3 males, 1 female, age: 24–36 yrs, height: 1.65–1.91 m, weight: 70–77.7 kg) wearing the exoskeleton, walking on a moving treadmill (see Figure 6A). The walking speed is set to 0.6 m/s speed due to limitations in the robot. We recorded muscle activities (electromyography (EMG) signals) of rectus femoris (REF), hamstring (HAM), medial gastrocnemius (GAS), and gluteus maximus (GLM). In addition, for the last two subjects, metabolics were measured (Oxycon Pro, Jaeger GmbH, Hoechberg, Germany) to assess how much energy expenditure can be reduced by the assistive controller. In this robot the knee and hip joints are active while the ankle is passive. All trials are compared to the transparent mode in which the robot follows the human subject's movements and tries to vanish the interaction force between human and the robot. More details about the experiments can be found in Zhao et al. (2017).

In this experiment the basic FMCH model is emulated by actuating the hip and knee actuators using the concept of biarticular muscles and the virtual leg. Since this controller is consistent with the VPP posture control concept (Sharbafi and Seyfarth, 2014, 2015) in human walking, the subjects feel comfortable with minimal opposing forces from the exoskeleton. This was quantitatively approved by measuring EMG signals and oxygen consumption. Figure 6B shows the reductions in muscle activation for different subjects. We compute the root mean square of the EMG signals for each trial subtracted by the EMG r.m.s. of the experiments performed in the transparent mode. It is observed that the average values for all muscles are positive meaning that this control technique can (in average) reduce the muscle activation of all four muscles. Concentration on different subjects' muscle activation also shows that the majority of the muscles are more relaxed (shown by positive numbers) in this assistive mode for every individual subject. For S2 (orange), all muscles have reduction in muscle activation. For S4 (green) HAM and GAS have significant reduction in EMG, while the two other muscle have small increase (not statistically significant) in muscle activation. For S3 (pink), all muscles are benefiting from assistance except HAM which shows increase in muscle activation. The results for the last subject is similar to S3, but with increase in REF instead of HAM. Thus, each subject benefits from assistance due to EMG reduction in majority of their muscles. The most assistance is observed in GAS muscle (positive for all subjects) and the least in REF muscle which might be related to their contributions in stance leg control. Furthermore, the results show that the robot and the subject interact with each other and the adaptation method varies between subjects. Using the ground reaction force (as a signal representing muscle reflexes) in adjusting the hip compliance makes the robot responsive to human reaction. This may also be a reason for different levels and patterns of assistance in different subjects.

Another measurable index for evaluating the performance of the controlled wearable robot is cost of transport. For this, we compare the oxygen consumption in two subjects (S3 and S4) with and without assistance (transparent mode). The results show 10.2% and 10.4% reduction in metabolic cost in the assistive mode compared to the transparent mode.

## 5. Discussion

In this paper, we introduced different studies preformed based on the concept of dividing legged locomotion to three sub-functions. Modeling and control of legged locomotion are challenged by nonlinearity, hybrid dynamics, uncertainties, dynamic coupling, etc. Dividing this complex problem to underlying sub-problems, helps better understand, design and control of legged locomotion. We have supported this hypothesis by several studies resulting in (i) acceptable prediction of different features in human gaits, (ii) stability analysis of the developed models and (iii) successful implementation on hardware setups such as BioBiped, MARCO-Hopper-II, and LOPES II. Therefore this new point of view in control can be a useful tool for managing the challenging legged locomotion problem.

In our studies, locomotor sub-function models are able to explain human gait in isolation and in interaction with each others both in conceptual models and detailed simulation model of robots. The stance and balance control approaches were also tested in experiments with MARCO II and LOPES II, respectively. In the studies on the BioBiped robot, we showed how stance and swing locomotor sub-functions can be synchronized if the posture is balanced. In this experiment the upper body was fixed by a frame as a physical constraint and in an exoskeleton, it can be performed by the a harness or another controller for balancing that assists (healthy or impaired) human subject. The proposed controller for swing leg is very simple, efficient and compatible with the FMCH based posture control when the leg switches from swing to stance. Therefore, the same mechanism (biarticular thigh muscles) and similar controller can be used between upper body and the leg for the swing and balance control.

In BioBiped experiments, motion speed adjustment by tuning the biarticular springs rest lengths provides a simple and efficient swing leg control approach with no need of sensory information from the leg configuration. In order to achieve high efficiency during different phases of the gait cycle (e.g., swing phase), non-backdrivable actuators are of advantage. They enable setting the springs' rest lengths to desired values, switching off the motors and operating with no (or little) resistance when no actuation is needed. Similar design and control can be employed to build an exoskeleton. The simple position control to set the biarticular springs' rest length via non-backdrivable motors can be implemented on soft exoskeletons (wearable robots) (Bartenbach et al., 2015).

The main idea of the proposed separate sub-function control in legged locomotion is the unifying control rule inspired from template models. Since the controllers use similar concepts of periodic movements through different oscillators, the sub-functions are harmonized by synchronizing the oscillators. Such an implicit coordination between different locomotor sub-functions is achieved via minimal sensory feedback. Roughly speaking, the spring in the SLIP model plays the role of a coordinator, the swing leg pendulum follows the CoM dynamics with fine tuning by hip springs and the force feedback used in FMCH synchronizes posture and stance control using the leg force which is generated by the leg spring. Hence, there is no need for extra higher level controller for synchronization of different sub-functions in steady state gait.

The built-in coordination with limited exchange of sensory information as the main advantage of distributed control using locomotor sub-functions, can be more visible in interaction with humans. In applications like control of wearable robots or prostheses, there is another high level supervisory controller from humans. Obviously, humans not only modify synchronization of different control level, by interacting with the robot, but also adapt to external torques and learn optimizing their efforts (as can be seen in the experiment with LOPES II). Indeed, reduction in energy consumption and muscle activation demonstrates that this adaptation improves support of human movement and the robot assists the human subject.

The next step of this research will be applying multiple (at least two) sub-functions controllers to the wearable robot (exoskeleton). For example, in LOPES II the BPDS and FMCH can be implemented for swing leg and balance control, respectively, through knee and hip actuation (the ankle which can be used for stance control is passive in this exoskeleton). In the recently developed wearable robot (called EMY) within the Balance project (http://balance-fp7.eu/), all three locomotor sub-functions can be controlled (with active hip, knee and ankle) and their interaction in assisting humans can be evaluated. In future, biomechanical gait templates should be extended to the sensor-motor level. Here, matching templates model could be identified taking actuator and sensor properties into account. With these models, additional design requirements for the different locomotor sub-functions both in biological and engineered systems can be derived.

## Author contributions

MS has the substantial contributions to the conception, drafting the work, final approval and is responsible for accuracy of the analysis and results. GZ has substantial contributions in design and performing experiments with LOPES II and analyzing the results. AS has also substantial contributions in design of the work, revising it critically for important intellectual content and final approval of the version to be published.

### Conflict of interest statement

The authors declare that the research was conducted in the absence of any commercial or financial relationships that could be construed as a potential conflict of interest.

## Footnotes

^1^PTS is the preferred speed for transition between running and walking which is typically about 1.9–2.1 m/s for humans (Lipfert, 2010).
